# B-mode and color Doppler ultrasonography of normal external jugular vein in donkeys (*Equus asinus*)

**DOI:** 10.1186/s12917-022-03441-y

**Published:** 2022-09-14

**Authors:** Hussein Awad Hussein, Ahmed Ibrahim

**Affiliations:** 1 Internal Veterinary Medicine, Department of Animal Medicine, Faculty of Veterinary Medicine, Assiut University, Assiut, 71526 Egypt; 2 Assistant Consultant of Surgery, Anesthesiology and Radiology, Veterinary Teaching Hospital, Faculty of Veterinary Medicine, Assiut University, Assiut, 71526 Egypt

**Keywords:** Equine, Doppler, Jugular vein, Measurements, Ultrasonography Thrombophlebitis

## Abstract

**Background:**

Although the jugular vein is a major important blood vessel in equine, the literature lacks this vessel's normal B-mode and Doppler ultrasonographic examinations in donkeys. Therefore, this study aimed to determine the reference ranges of B-mode and Doppler ultrasonographic indices of jugular veins in healthy adult donkeys (*Equus asinus*) and the possible effect of examination side (left and right), gender, and body condition on the ultrasonographic measurements of this vessel. B-mode and Doppler ultrasound imaging of the external jugular vein was conducted on 20 adult healthy donkeys of both sexes.

**Results:**

In all donkeys, the jugular vein was 4.01 to 8.1 mm from the body surface. The longitudinal and transverse venous diameters ranged from 3.94 to 10.5 mm and from 0.88 to 1.9 cm, respectively. Moreover, the vein areas varied from 0.61 to 2.83 cm^2^. The reference values of superficial and deep wall thickness (SWT and DWT) were 0.56 ± 0.2 and 0.6 ± 0.13 mm, respectively. The blood velocity, blood follow rate, and congestion index of the external jugular vein can be expected in adult healthy donkeys as a range value from 8.4 to 13.5 cm/sec, from 0.33 to 1.78 ml/min, and from 0.06 to 0.27 cm.sec, respectively. Generally, the vein showed laminar monophasic waveforms. The examination side and gender have no significant effect on the ultrasound measurements of the vein (*P* > 0.05). Donkeys with a body condition score (BCS) ≥ 3 revealed increases in the depth of the vein (*P* < 0.05).

**Conclusions:**

The results of this study can be used as reference values and provide a basis for comparison when evaluating donkeys with diseases that affect blood flow in the external jugular vein.

## Background

The vein is a thin-walled vessel that carries blood back to the heart. Jugular veins are formed by the union of the maxillary and linguofacial veins and are located in the right and left jugular furrow of the neck sides in equines [1]. The jugular vein is the most common site of venipuncture for blood sample collections, intravenous medications, or intravenous catheterization [2–4]. Therefore, it is commonly exposed to vascular-related diseases such as phlebitis, thrombophlebitis, or venous embolism [3, 5–9].

Early detection of jugular vein disorders is valuable to provide proper treatment and prevent further worse consequences such as thrombotic vein occlusion, head swelling with subsequent respiratory distress, or organ infarction from released emboli [3, 4, 10].

Ultrasonography is a reliable diagnostic tool for the early detection or progression of jugular vein abnormalities by detecting the vascular wall thickness and luminal patency [3, 11]. Moreover, Doppler ultrasonography is an essential non-invasive and safe clinical tool commonly used in the diagnosis of vascular diseases in humans [12] and horses [13]. Additionally, Doppler ultrasonography could be used as a follow-up tool during the treatment and monitoring of vascular diseases. It also allows simultaneous scanning and spectral analysis of vascular flows [14]. Both qualitative and quantitative information could be obtained during the assessment of these flow patterns concerning a particular vascular disease [15]. B-mode ultrasonographic examination of the jugular vein has been documented in horses [11].

Neck anatomical differences between donkeys (*Equus asinus*) and horses (*Equus caballus*) have been documented in the literature [16–18]. In donkeys, the cutaneous muscle of the neck (cutaneous colli) covers the entire external jugular vein within the jugular groove, while in horses, this muscle is in the lower or caudal 1/2 to 1/3 of the neck. Still, it seldom covers the entire external jugular vein in the neck region. This muscle's extensive nature tenses the neck's skin in donkeys [16, 17]. Moreover, donkeys have thick skin [19]. Such variation between donkeys and horses was the motivation for the conduction of this study.

However, to the best of the authors' knowledge, there is no literature reporting the normal B-mode and Doppler ultrasonographic examinations of external jugular veins in donkeys. Therefore, the objectives of this study were as follows: (1) determine the reference ranges of B-mode and Doppler ultrasonographic indices of jugular veins in clinically healthy adult donkeys (*Equus asinus*); (2) determine the possible effect of examination site, sex, and body condition on the ultrasonographic measurements of the jugular vein.

## Results

### Findings of body condition scoring (BCS)

The body condition score (BCS) of donkeys ranged from 2 to 4. Eight donkeys with BCS ≥ 3 and twelve donkeys with BCS < 3 were found in the current study.

Donkeys with a condition score of 2 showed the following: few muscles over dorsal withers; spinous processes were obvious but not prominent; ribs not visible but can be felt easily with palpation; dorsal and transverse processes felt with light palpation; poor muscle deposition over both sides of the midline; poor muscle cover on hindquarters; hipbones felt with palpation.

Donkeys with a BCS of 3 exhibited the following: developed Superspinous muscles; vertebral column can be felt with palpation; little amounts of fat can be felt in the base of the neck and shoulder area; ribs covered by light muscles; rounded hooks and rumps; pins were not visible; and good muscle covers hind quarters.

Donkeys with a score of 4 revealed the following: difficult to detect spinous processes during palpation; the back was flat and well covered; rump convex and well-muscled; good fat felt on the neck and shoulder area; neck filled into the shoulder; broad withers; ribs felt with deep palpation; and rounded hind quarters with evenly distributed fat deposits.

### Clinical and laboratory findings

All animals were clinically healthy during the study period. The mean values of rectal temperature, heart, and respiratory rates were 37 ± 2.3 ºC, 39 ± 3.4 beats/min, and 15 ± 1.2 cycles/min, respectively. Abdominal auscultation revealed 2.4 ± 0.5 borborygmi/min. Oral mucous membranes were moist and pink in color, and the mean value of capillary refilling time was 1.6 ± 0.04. No signs of coagulopathies or cardiovascular diseases were observed. Neither jugular pulsation nor distention was noticed during the study period. Heart auscultation revealed neither cardiac murmurs nor arrhythmias. The mean values of PT and APTT were 9.3 ± 1.3 and 26 ± 2.4 s, respectively.

### B-mode and color Doppler ultrasonography of the jugular vein

**Fig. 1:**
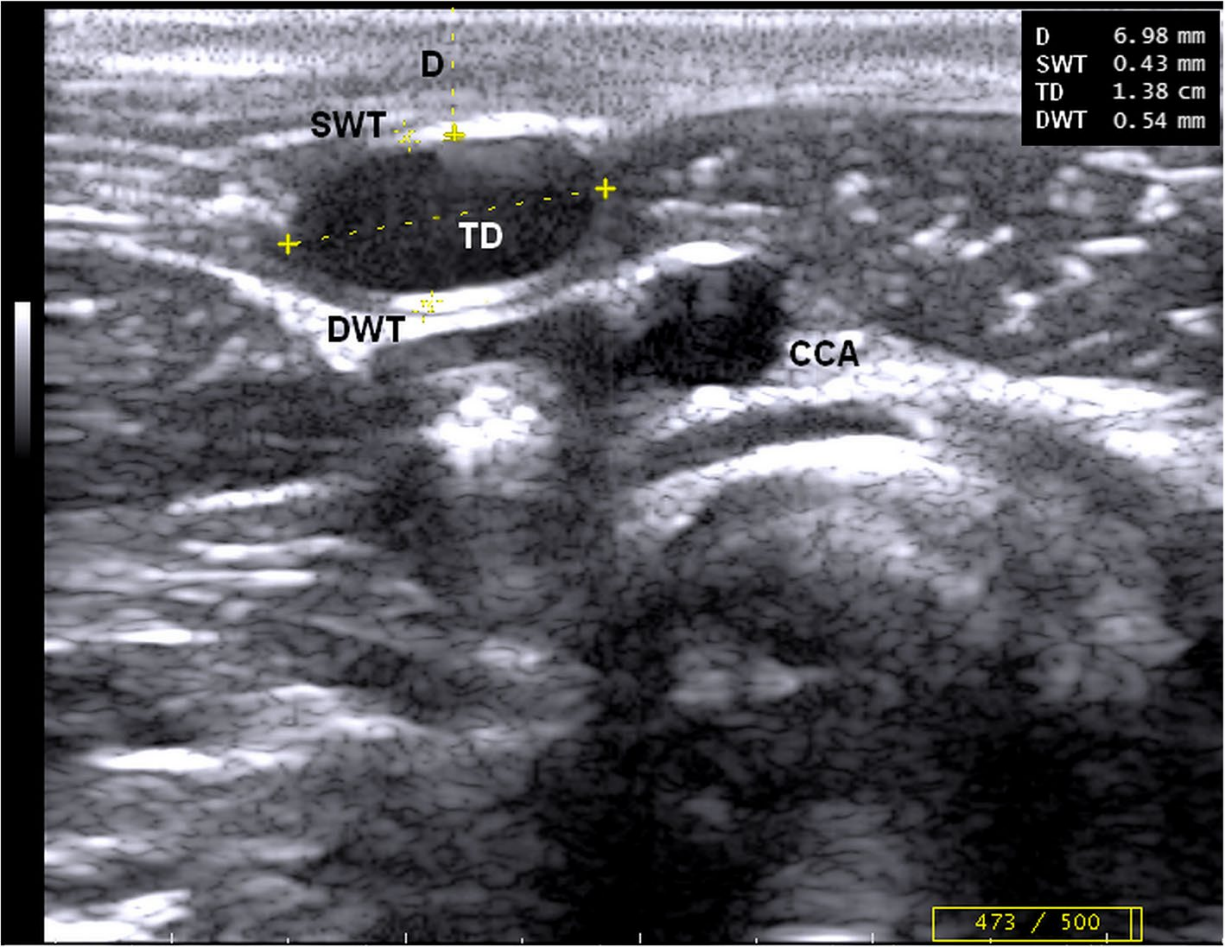
B-mode ultrasonographic image in the transverse plane of the right jugular vein in a donkey at the middle of the neck shows the depth (D), superficial wall thickness (SWT), longitudinal diameter (LD), and deep wall thickness (DWT)

**Fig. 2:**
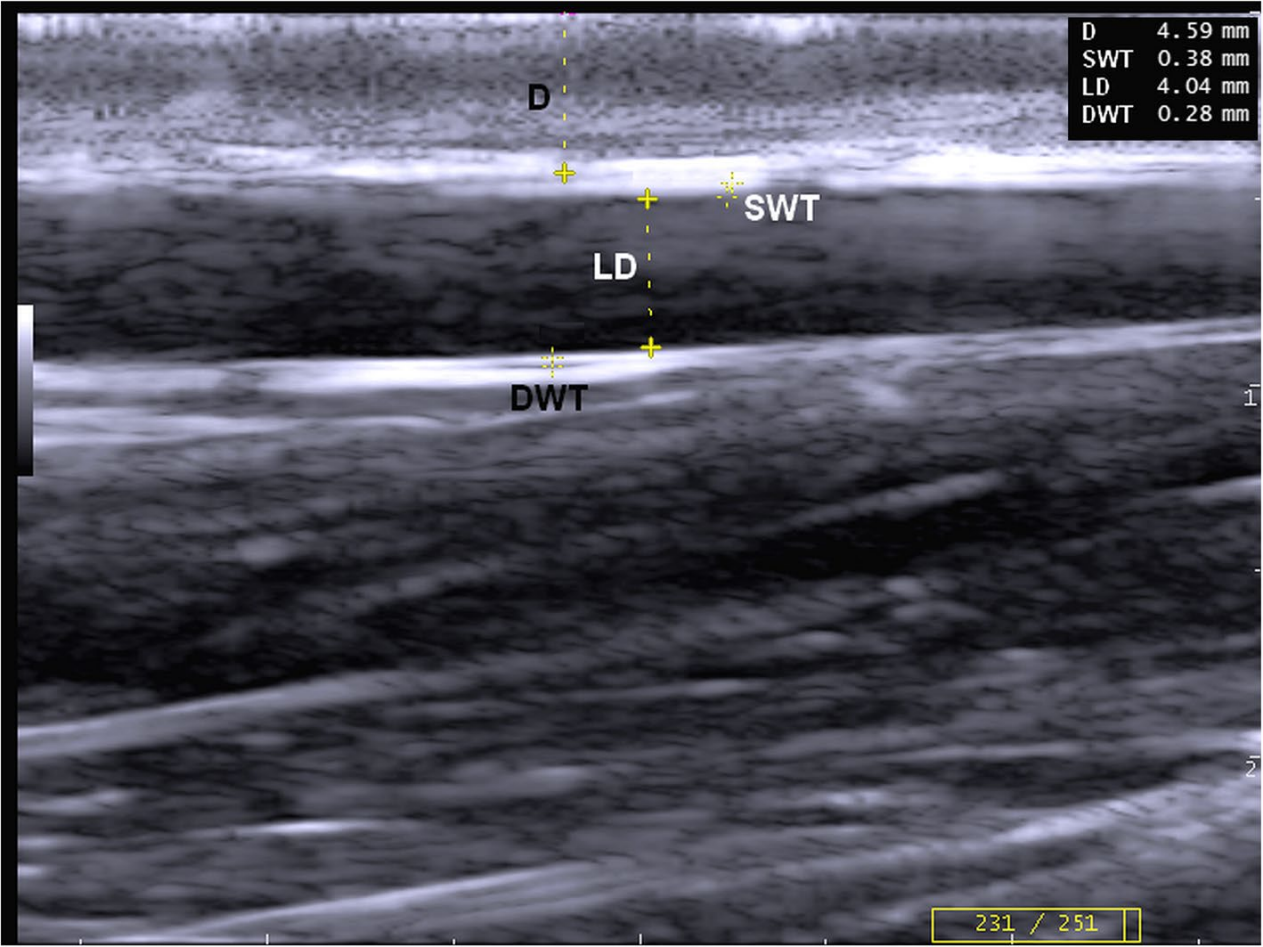
B-mode ultrasonographic image in the longitudinal plane of left jugular vein in a donkey at the middle third of the neck shows the depth (D), superficial wall thickness (SWT) longitudinal diameter (LD), and deep wall thickness (DWT)

**Fig. 3:**
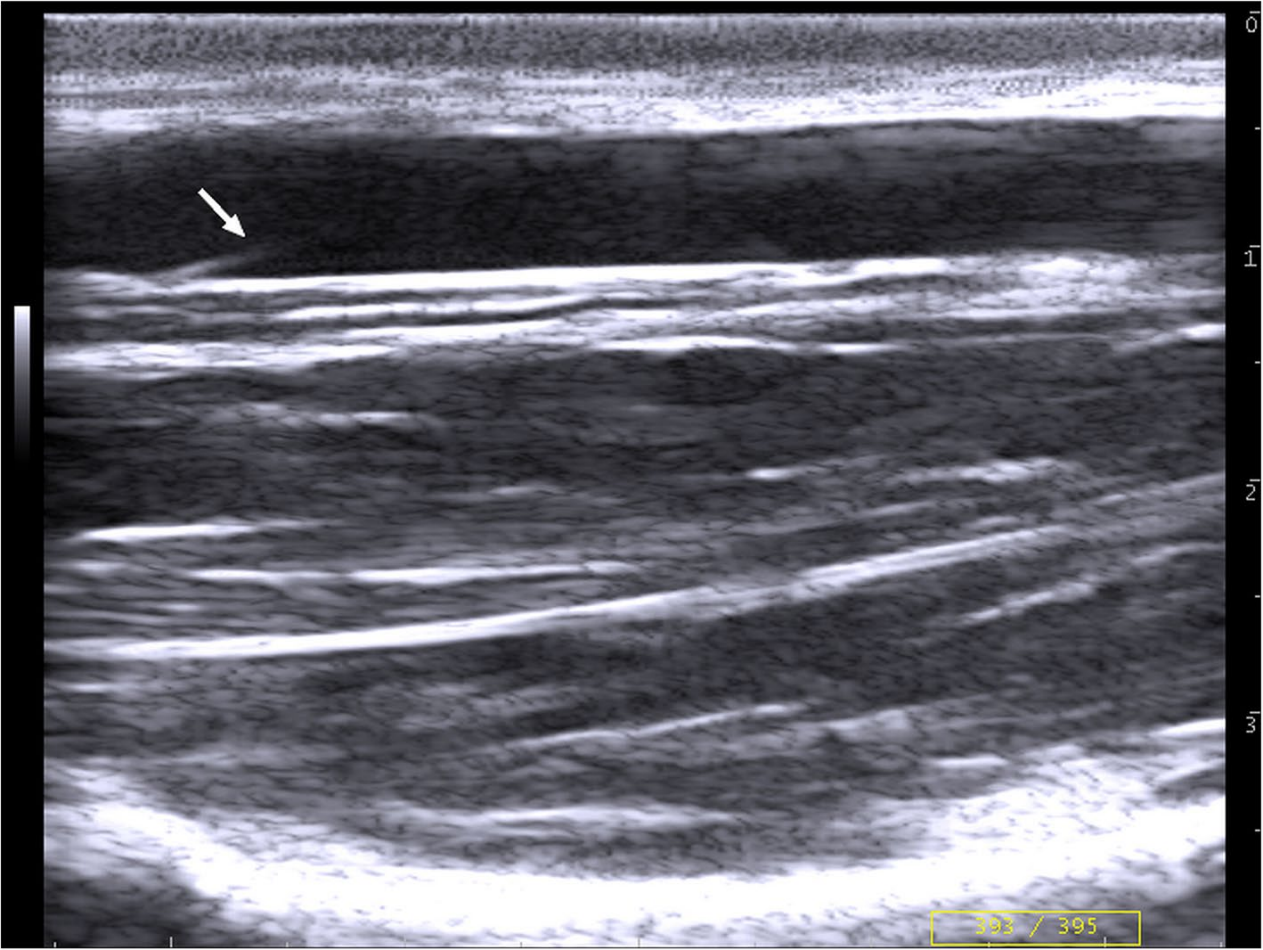
B-mode ultrasonographic image in the longitudinal plane of the right jugular vein in a donkey at the middle third of the neck shows a vein valve (arrow)

In all donkeys, the jugular vein, using B-mode ultrasonography, was visualized as an anechoic tubular structure, representing the blood, with thin echogenic walls immediately beneath the skin (Figs. 1 & 2). The vein was 4.01 to 8.1 mm from the body surface. Jugular vein valves were also visualized as hyperechoic structures, and their rhythmic movement further signifies blood flow and therefore venous patency (Fig. 3).

**Fig. 4:**
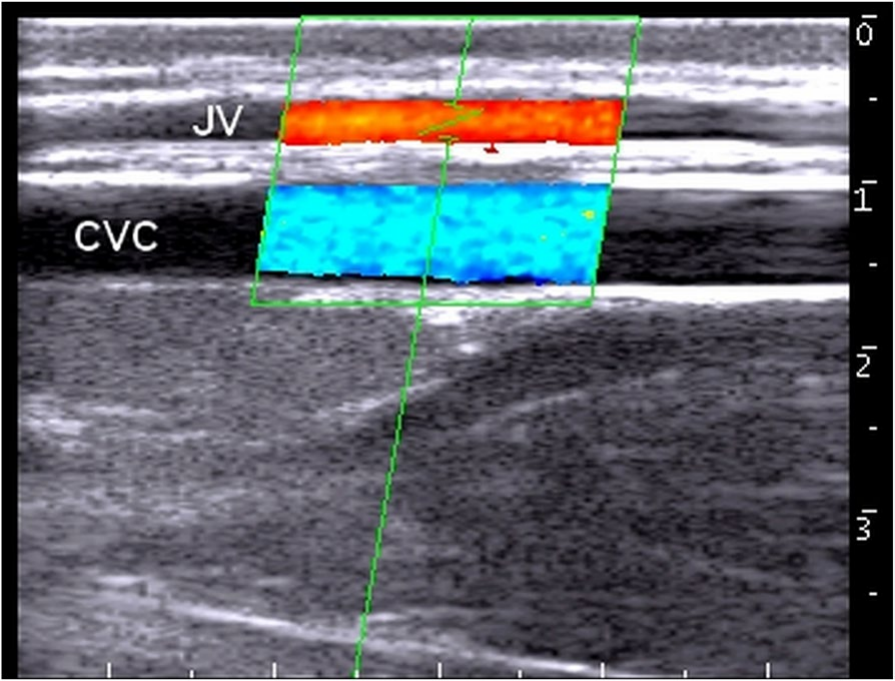
Color Doppler scan of the right jugular vein (JV) and common carotid artery (CCA) at the middle third of the neck

Using color Doppler ultrasonography, tracing the blood flow in the jugular vein revealed the direction toward the thoracic inlet. The wall of the vein was thin and smooth with consistent color fill, indicating absence of color filling defects (Fig. 4). Furthermore, no pulsatility, aliasing, or vein deformity was seen, as well as no collateral vessels were imaged.

**Fig. 5:**
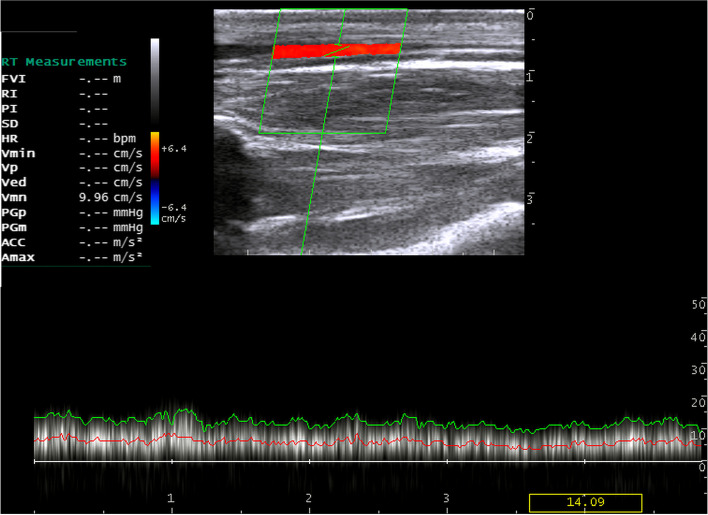
Color Doppler image for the left jugular vein at the middle third of the neck. Note: The spectral waveform shows smooth laminar blood flow with a mean velocity of 9.96 cm/s

**Fig. 6:**
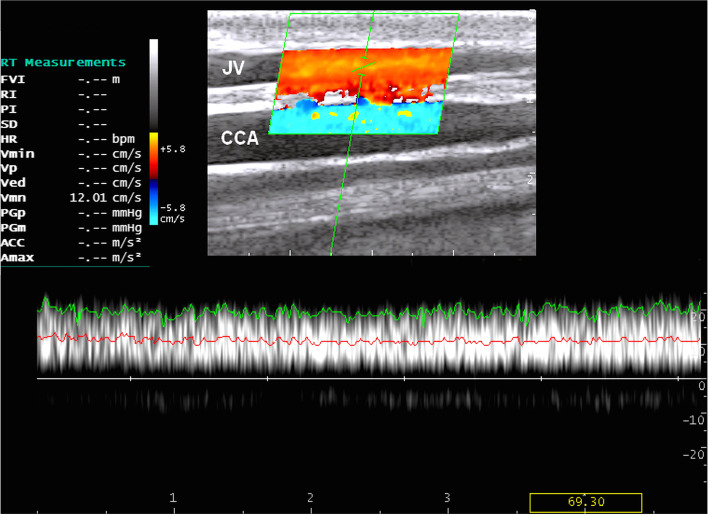
Triplex ultrasound scan of the left jugular vein at the upper third of the neck with a mean blood velocity of 12.01 cm/s. The spectral waveform shows turbulent blood flow. JV, Jugular vein; CCA, common carotid artery

**Fig. 7:**
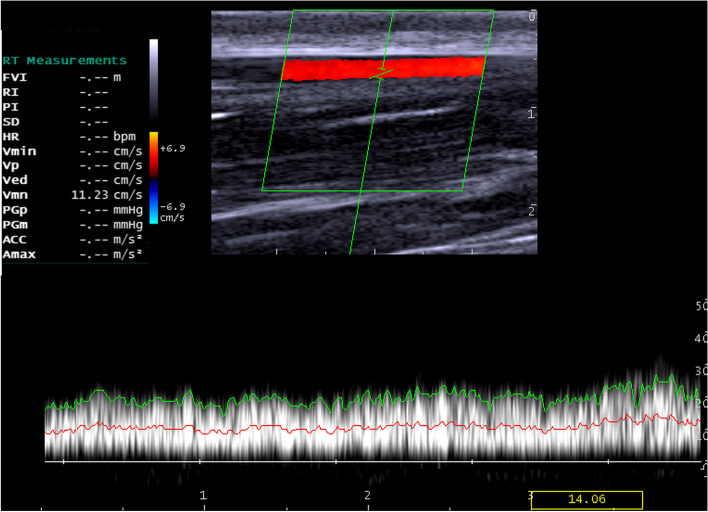
Spectral Doppler scan of the right jugular vein at the lower third of the neck with a mean blood velocity of 11.23 cm/s. The spectral waveform shows turbulent blood flow

Triplex ultrasonographic scans of the jugular vein revealed no evidence for the presence of flow disturbance and incompetence, indicating no venous reflux. In the middle of the vein, the spectral waveforms showed monophasic laminar blood flow (Fig. 5), and the blood velocities ranged from 8.4 to 13.5 cm/sec. However, at the thoracic inlet and mandibular angle, Doppler hemodynamics of the jugular vein revealed monophasic waveforms with turbulent blood flows (Figs. 6 & 7), and the blood velocities ranged from 10.3 to 15.2 cm/sec. In addition, the jugular veins of both sides were completely compressible with minimal pressure on the probe. Furthermore, the spectral waveform analysis of jugular veins showed the respiratory phasicity in 7 donkeys.

**Fig. 8:**
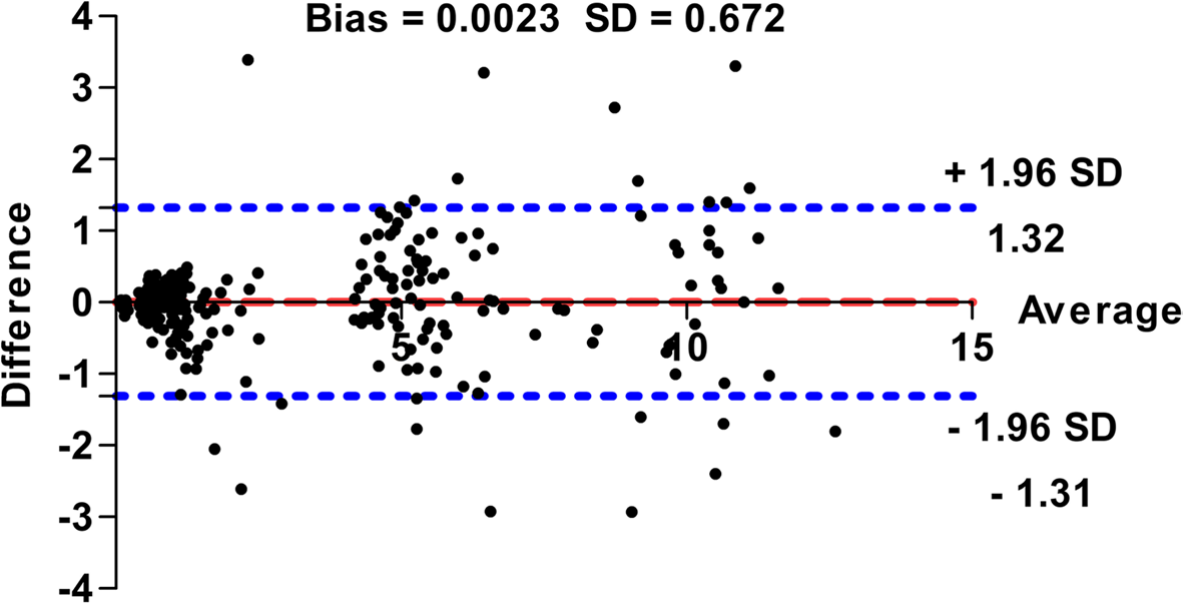
Bland–Altman difference plot for ultrasonographic measurements from the left and right sides of the jugular vein in donkeys. Y-axis: The ultrasound measurements taken from the left side minus the value of the same measurements obtained from the right one (Difference). X-axis: Average of the ultrasound measurements taken from the left and right sides. The mean of the differences or bias (red dashed line) and the 95% limits of agreement (mean ± 1.96 SD) are included in the graph (blue dotted lines)

Table 1 summarizes B-mode and Doppler ultrasound measurements including the vein depth (D), superficial wall thickness (SWT), deep wall thickness (DWT), longitudinal and transverse diameters, blood velocity, blood flow rate (BFR), and congestion index (CI) with normal reference values for the left and right jugular veins and interclass correlation coefficients (ICC). The ICC exhibited good-to-excellent measurement repeatability. There were no significant changes between mean left and right ultrasound measurements over the 3 consecutive days in 20 normal donkeys (*P* > 0.05). The reproducibility of the jugular vein depth was excellent at left side (*P* = 0.53; ICC = 0.82) and good at right side (*P* = 0.58; ICC = 0.78) during longitudinal scans, while it was good (left, *P* = 0.47; ICC = 0.73 and right, *P* = 0.42; ICC = 0.79) with transverse views. Over the 3 consecutive days, there were no significant variations between mean left and right vein diameters (*P* > 0.05). The reproducibility of the longitudinal jugular diameters was excellent (*P* = 0.055; ICC = 0.80) for left veins and good (*P* = 0.57; ICC = 0.77) for right ones. The reproducibility of the transverse jugular vein diameters was good (left, *P *= 0.44; ICC = 0.76 and right, *P* = 0.51; ICC = 73). Moreover, good reproducibility was observed for the jugular vein areas (left, *P* = 0.33; ICC = 0.75 and right, *P* = 0.37; ICC = 0.73). Furthermore, there were no significant differences between left and right Doppler ultrasonographic measurements of jugular vein over 3 consecutive days (*P* > 0.05). The reproducibility was good for blood velocity (left, *P* = 0.39; ICC = 0.72 and right, *P* = 0.32; ICC = 0.76) and blood flow rate (left, *P* = 0.42; ICC = 0.71 and right, *P* = 0.30; ICC = 0.79) and congestion index (left, *P* = 0.41; ICC = 0.73 and right, *P* = 0.31; ICC = 0.76). Table 2 shows the comparison between left and right ultrasonographic measurements of the jugular vein. The examination side exhibited non-significant effect on B-mode and Doppler measurements of the jugular vein (*P* > 0.05). Moreover, Bland–Altman plot indicated small bias between left and right sides’ measurements (bias = 0.0023, SD = 0.672; Fig. 8), and 95% limits of agreements were also included, indicating the association between left and right-side measurements.

**Table 1:** Normal reference values (mean ± 2 SD), minimum and maximum measurements and ICCs for ultrasonographic scans of jugular veins in donkeys (*Equus asinus*, *n* = 20)

	Left side veins				Right side veins			
	Reference values	Minimum	Maximum	ICCs	Reference values	Minimum	Maximum	ICCs
Longitudinal scans								
Depth (mm)	5.3 ± 1.9	4.1	8.1	0.82	5 ± 1.4	4.1	6.8	0.78
SWT (mm)	0.58 ± 0.2	0.42	0.89	0.78	0.57 ± 0.12	0.45	0.68	0.81
DWT (mm)	0.6 ± 0.2	0.45	0.87	0.75	0.61 ± 0.17	0.42	0.77	0.80
Diameter (mm)	5.9 ± 2.3	4.2	10.10	0.80	6 ± 3.4	3.94	10.5	0.77
Transverse scans								
Depth (mm)	5.5 ± 1.6	4.01	7.7	0.73	5.4 ± 2.7	0.62	7.79	0.79
SWT (mm)	0.56 ± 0.2	0.43	0.79	0.84	0.59 ± 0.15	0.45	0.77	0.72
DWT (mm)	0.6 ± 0.2	0.43	0.77	0.77	0.6 ± 0.13	0.5	0.7	0.75
Diameter (cm)	1.1 ± 0.4	0.88	1.64	0.76	1.2 ± 0.56	0.91	1.9	0.72
Area (cm^2^)	1.1 ± 0.5	0.61	2.11	0.75	1.2 ± 0.6	0.65	2.83	0.73
Doppler scans								
Blood velocity (cm/sec)	10.5 ± 2	8.4	12.5	0.72	10.4 ± 2.2	8.5	13.5	0.76
Blood flow rate (ml/min)	0.67 ± 0.3	0.36	1.39	0.71	0.77 ± 0.4	0.33	1.78	0.79
Congestion index (cm.sec)	0.12 ± 0.08	0.06	0.19	0.73	0.11 ± 0.06	0.06	0.27	0.76

*Abbreviations*: *SD* Standard deviation, *ICCs* Interclass correlation coefficients for measurements of repeatability

**Table 2:** Effect of examination side (left and right) on B-mode and Doppler ultrasonographic measurements of jugular vein in donkeys (*Equus asinus*, *n* = 20)

	B-mode scans									Doppler scans		
	Longitudinal views				Transverse views					Blood velocity (cm/sec)	Blood flow rate (ml/min)	Congestion index (cm.sec)
	Depth (mm)	SWT^a^ (mm)	DWT^b^ (mm)	Diameter (mm)	Depth (mm)	SWT^a^ (mm)	DWT^b^ (mm)	Diameter (cm)	Area (cm^2^)			
Left (*n* = 20)	5.3 ± 0.2	0.58 ± 0.02	0.60 ± 0.02	5.9 ± 0.3	5.5 ± 0.3	0.56 ± 0.02	0.6 ± 0.02	1.1 ± 0.04	1.1 ± 0.1	10.5 ± 0.2	0.67 ± 0.06	0.12 ± 0.01
Right (*n* = 20)	5.0 ± 0.1	0.57 ± 0.01	0.61 ± 0.01	6.0 ± 0.3	5.4 ± 0.2	0.59 ± 0.01	0.6 ± 0.01	1.2 ± 0.05	1.2 ± 0.1	10.4 ± 0.2	0.77 ± 0.07	0.11 ± 0.01
*P*-value	0.283	0.564	0.701	0.981	0.518	0.107	0.759	0.291	0.264	0.578	0.388	0.172

Data are presented as mean ± standard error

^a^ *SWT* Superficial wall thickness

^b^ *DWT* Deep wall thickness

The mean values of B-mode and Doppler measurements showed no significant variation between male and female donkeys (*P* > 0.05) (Table 3), indicating that gender has no great influence on the ultrasonographic measurements of the jugular vein. Table 4 shows the effect of body condition on the mean ultrasound measurements of the jugular vein. Donkeys with body condition score (BCS) ≥ 3 revealed increases in the depth of the vein (*P* < 0.05).

**Table 3:** Effect of gender on B-mode and Doppler ultrasonographic measurements of jugular vein in donkeys (*Equus asinus*, *n* = 20)

	B-mode scans									Doppler scans		
	Longitudinal views				Transverse views					Blood velocity (cm/sec)	Blood flow rate (ml/min)	Congestion index (cm.sec)
	Depth (mm)	SWT^a^ (mm)	DWT^b^ (mm)	Diameter (mm)	Depth (mm)	SWT^a^ (mm)	DWT^b^ (mm)	Diameter (cm)	Area (cm^2^)			
Male (*n* = 10)	5.3 ± 0.2	0.5 ± 0.01	0.6 ± 0.01	6.5 ± 0.2	5.6 ± 0.2	0.6 ± 0.01	0.6 ± 0.01	1.1 ± 0.04	1.1 ± 0.08	10.5 ± 0.2	0.7 ± 0.05	0.10 ± 0.01
Female (*n* = 10)	4.9 ± 0.1	0.5 ± 0.01	0.6 ± 0.02	6.7 ± 0.4	5.0 ± 0.1	0.6 ± 0.01	0.6 ± 0.01	1.2 ± 0.06	1.3 ± 0.1	10.4 ± 0.2	0.8 ± 0.09	0.12 ± 0.01
*P*-value	0.133	0.843	0.607	0.076	0.078	0.845	0.882	0.156	0.177	0.912	0.188	0.177

Data are presented as mean ± standard error

^a^ *SWT* Superficial wall thickness

^b^ *DWT* Deep wall thickness

**Table 4:** Effect of BCS on B-mode and Doppler ultrasonographic measurements of jugular vein in donkeys (*Equus asinus*, *n* = 20)

	B-mode scans									Doppler scans		
	Longitudinal views				Transverse views					Blood velocity (cm/sec)	Blood flow rate (ml/min)	Congestion index (cm.sec)
	Depth (mm)	SWT^1^ (mm)	DWT^2^ (mm)	Diameter (mm)	Depth (mm)	SWT^1^ (mm)	DWT^2^ (mm)	Diameter (cm)	Area (cm^2^)			
BCS ≥ 3 (*n* = 8)	5.4 ± 0.2^a^	0.6 ± 0.02	0.6 ± 0.02	6 ± 0.4	5.8 ± 0.2^a^	0.57 ± 0.02	0.6 ± 0.01	1.2 ± 0.04	1.2 ± 0.1	10.7 ± 0.2	0.8 ± 0.1	0.1 ± 0.01
BCS < 3 (*n* = 12)	4.9 ± 0.1^b^	0.5 ± 0.01	0.6 ± 0.02	5.8 ± 0.2	5.1 ± 0.2^b^	0.58 ± 0.01	0.6 ± 0.01	1.1 ± .04	1.1 ± 0.1	10.2 ± 0.2	0.7 ± 0.1	0.1 ± 0.01
*P*-value	**0.025**	0.195	0.834	0.613	**0.014**	0.925	0.793	0.531	0.579	0.091	0.420	0.800

Data are presented as mean ± standard error

^1^ *SWT* Superficial wall thickness

^2^ *DWT* Deep wall thickness

^ab^ means in the same column differ significantly *P* < 0.05

## Discussion

The jugular vein is a major important blood vessel in equine because it is the most common site used for collecting of blood samples, intravenous injections, and catheterization [20, 21]. Therefore, many diseased conditions have been noticed in such vein including phlebitis and thrombophlebitis [22, 23], and aneurysm [13].

As mentioned in previous studies in horses [5, 22], many complications may develop because of jugular thrombophlebitis including impaired venous drainage of the head with subsequent brain congestion or edema and impaired thermoregulation, congestion, or edema of the nasal, pharyngeal, or laryngeal mucosa that causes dysphagia and/or dyspnea. Horses with jugular vein aneurysm are usually presented with a compressible asymptomatic cervical mass, enlarged upon manual compression of the vein [13].

Ultrasonography is necessary for the diagnosis and differential diagnosis of jugular vein diseases in horses, and the determination of the extent and severity of lesions [23, 24]. Furthermore, information obtained from ultrasonographic examination of the jugular vein can be used to assess central venous pressure, which may provide useful data about intravascular-volume status, adequacy of fluid resuscitation, presence of congestive heart failure, or other abnormal conditions, such as jugular phlebitis and thrombophlebitis, and pericardial tamponade [3, 25]. Moreover, Doppler ultrasound imaging confirmed jugular vein aneurysm in horses [13].

The present study has provided the researchers and veterinarians with the reference ranges for B-mode and Doppler ultrasound measurements of the jugular vein in adult healthy donkeys. Based on the results of this research, longitudinal venous diameters ranged from 3.94 to 10.5 mm, transverse venous diameters from 0.88 to 1.9 cm, and area from 0.61 to 2.83 cm^2^. The values of SW and DW thicknesses can be expected as a range from 0.42 to 0.89 and from 0.42 to 0.87 mm, respectively, in adult healthy donkeys. The reliability of the ultrasonographic measurements was excellent to good, indicating good repeatability. Unfortunately, no literature studies are available reporting the normal ultrasonographic measurements of jugular vein in donkeys to discuss the present study results with. However, B-mode ultrasound measurements in the current work were lower than those reported in horses [11], where the authors reported higher values for jugular vein depth, diameter, SWT and DWT, and area. Such differences could be explained by species and/or anatomical variations.

This study provides the clinicians, for the first time, with the reference Doppler ultrasound measurements of the jugular vein in donkeys. Based on the present results, blood velocity, blood flow rate, and congestion index ranged from 8.4 to 13.5 cm/sec, from 0.33 to 1.78 ml/min, and from 0.06 to 0.27 cm/sec, respectively, in adult healthy donkeys. Unfortunately, according to the authors’ knowledge, no reference values for Doppler ultrasonography of jugular veins in donkeys or horses to compare the current results. However, the values of this study seem to be lower than those reported in cows [26], which could be explained by species variation. In the current research, Doppler hemodynamics of the jugular vein revealed monophasic waveforms with laminar smooth blood flows at the middle of the vein pathway, in contrast, turbulent flows were detected at the thoracic inlet and mandibular angle. Such differences could be attributed to increased blood velocities in the jugular vein near the mandibular angle and thoracic inlet. Turbulent blood flows occur when the cross-sectional area of the bloodstream suddenly changes with subsequently increased blood velocities [27]. Moreover, in this current study, jugular veins were compressible with minimal pressure on the probe, indicating patency and no evidence for thrombosis. As reported in a previous study [28], loss of vein compressibility during scanning is a good indicator of the presence of a thrombus within the vein. Furthermore, the spectral waveform analysis, in this work, showed the respiratory phasicity, indicating a decrease in venous flow with inspiration and an increase with expiration. As reported elsewhere [29], the authors attributed the respiratory phasicity of venous flows to the variations in intrathoracic pressure during respiratory movements.

In this work, the ultrasound measurements obtained from the right and left jugular veins did not differ, as well as the ultrasound indices of both sides were associated with small bias, indicating the examination side has no significant influence on the measurements. This finding indicates no substantial variation between the measurements of the left and right sides. Consequently, using either the left or right examination side is feasible for the evaluation of jugular veins in healthy donkeys. In a previous study on Standardbred horses [11], the authors found no significant changes for ultrasound measurements between the left and right sides, except for the dorsoventral diameter in the longitudinal scan, which significantly increased in the left veins. In contrast, little variations were determined between the right and left external jugular veins in cows [26]. In the current research, no significant changes were noticed between male and female animals, indicating that gender has no significant influence on the ultrasound measurements of jugular veins. This finding was supported by a previous study in horses [11]. In contrast, the cross-sectional areas of internal jugular veins were significantly influenced by gender in healthy humans [30]. In this work, the depth of the jugular vein increased with increased body condition, which could be explained by increased subcutaneous fat in obese donkeys. In a previous study in human medicine [31], the authors supported the potential role of adipose tissues in the variation of ultrasound measurements of lower limb veins between healthy obese and nonobese individuals. Although the number of donkeys, in the current study, was somewhat limited, the obtained results are promising as a starting point and suggest the conduction of a further study including a greater number of animals.

## Conclusions

The current study presented the normal values of B-mode and Doppler ultrasonographic measurements for the external jugular vein in adult healthy donkeys (*Equus asinus*). The results of the study reported here can be used as reference values, and provide a basis for comparison when evaluating donkeys with diseases that affect blood flow in the external jugular vein. The examination side and gender do not affect the ultrasound measurements of the jugular vein. Moreover, the more body condition score, the more depth of external jugular vein in donkeys.

## Methods

### Animals and study design

This study was ethically approved by the Animal Care and Welfare Committee of the Faculty of Veterinary Medicine, Assiut University, Assiut, Egypt. All national and institutional guidelines and rules for the care and use of animals were followed during the research procedures. All methods were performed according to the guidelines and regulations. All animals were housed and cared according to the Egyptian animal welfare act (No. 53, 1966). The study was conducted on 20 adult clinically healthy donkeys (*Equus asinus*), of both sexes (10 males, and 10 non-lactating and non-pregnant females). The donkeys were collected from those animals belonging to the veterinary teaching hospital, Assiut University. The average age was 6 ± 1.5 years, and weight was 107 ± 3.3 kg (mean ± standard error). No history of intravenous injection in the jugular veins in the last 5 months. All donkeys were examined clinically [32]. Donkeys showing any clinical and/or cardiovascular abnormalities were removed from the study. All donkeys were housed in a free stable yard with feed and water *ad libitum*.

### Evaluation of body condition scoring

Using visual inspection and palpation, the body condition score (BCS) for each donkey was evaluated using a 1 – 5 scale according to the chart previously described by Burden [33].

### Clinical examination, blood sampling, and laboratory analysis

To assure the health status and exclude the diseased donkeys, all animals were examined clinically, and their blood samples were tested in the laboratory.

The general condition, mental status, appetite, defecation, urination, rectal temperature, heart and respiratory rates, and intestinal motility were assessed in the initial physical examination. A brief clinical examination, including inspection and palpation of the jugular veins, was carried out before each ultrasonographic examination.

From each donkey, a blood sample was collected on a tube containing sodium citrate as an anticoagulant. Then, plasma samples were harvested for estimation of coagulation profiles including prothrombin time (PT) and activated partial thromboplastin time (APTT) using commercial test kits (BIO MED DIAGNOSTICS, Germany).

### Jugular ultrasonographic examination

#### Animal preparation

All ultrasonographic examinations were conducted in a standing position without sedation. For examination, donkeys were kept in the stock and restrained by an equine handler. In each donkey, the area over the jugular grooves within the entire length of the neck on both sides was shaved, washed, and disinfected with 70% alcohol.

#### B-mode and Doppler sonographic examination

**Fig. 9:**
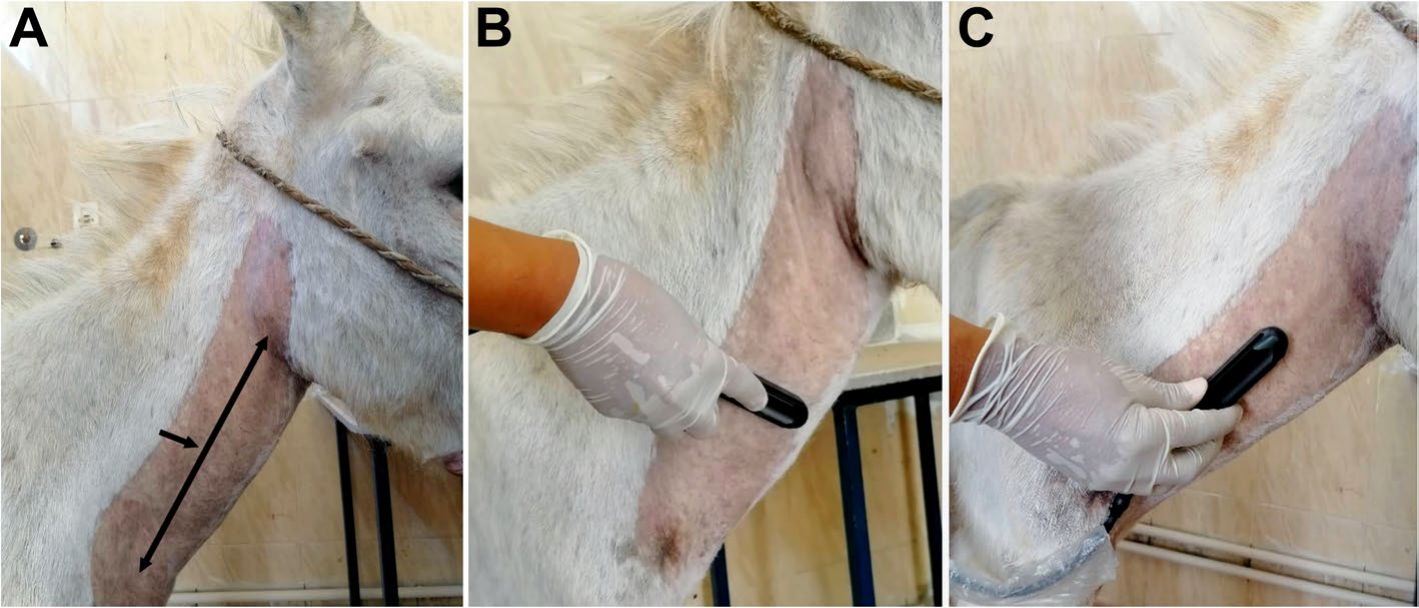
The midway between the mandibular angle and thoracic inlet was taken as a standard point for ultrasound measurements of the jugular vein (**A**). For ultrasound probe positions, firstly the probe was placed in a transverse plane (**B**), then the probe was redirected to be in a longitudinal plane (**C**). All ultrasonographic measurements were taken in both planes

The procedures of B-mode and Doppler scans were performed by the same operator using a multi-frequency linear probe (6–10 MHz, MyLabOne VET, Esaote, Italy). Caution was taken not to compress the jugular vein during the examination. To detect any abnormal vein, firstly the jugular veins were scanned within their whole length in the jugular furrow. However, all measurements for B-mode and Doppler scans were taken midway between the mandibular angle and thoracic inlet (Fig. 9a). Obtaining ultrasound measurements were standardized to be at 08:00 before the morning feedings.

Using B-mode, the veins were examined in cross-section to locate the vein of interest (Fig. 9b), then longitudinal scan with the transducer surface positioned parallel to the jugular vein (Fig. 9c). At the widest distance, the internal diameters of the jugular veins (i.e., intimal surface-to-intimal surface) (transverse diameter, TD) were measured electronically in cross-section scan after freezing the ultrasonographic image, then vein areas were calculated using the following equation [34]:

$$\mathrm A=\left(\mathrm D^2\mathrm x\;\mathrm\pi\right)/4$$

where A: vein area; D: vein diameter; π: 3.14.

In transverse and longitudinal scans, the distance from the skin to the intimal surface of the vein was measured as the vein depth (D). Moreover, superficial wall thickness (SWT) and deep wall thickness (DWT) were measured as the distance from the leading edge of the lumen-intima interface of the wall to the leading edge of the media-adventitia interface of the wall (Figs. 2 & 3) [35]. Furthermore, longitudinal diameters (LD) were also measured (Fig. 3).

For Doppler ultrasonographic examination, default adjustments of the ultrasound device for the superficial parts were done. In longitudinal scans, color and spectral Doppler examination was carried out for jugular veins to evaluate blood flow direction and velocity within the vessel. Doppler sample size was adjusted to incorporate the two-third of the vein. The angle between the sound waves and the blood flow direction in the jugular vein was kept to be less than 60 degrees in all Doppler scans. Doppler ultrasonographic examinations of jugular veins were performed within the entire length of the jugular furrows, however, the measurements were taken in the middle between the mandibular angle and thoracic inlet. In all donkeys, Doppler spectral waveforms were recorded at a pulse repetition frequency (PRF) that was sufficient to prevent aliasing artifacts [36]. Moreover, the mean blood flow velocity (Vmean) was assessed through the uniform insonation technique by the device; the angle correction was used in all spectral scans to measure the velocities accurately [37].

The blood flow rate (BFR) and the congestion index (CI) were evaluated according to the obtained data of the vein studied. To calculate BFR of the jugular vein, the following formula was used as mentioned by Kantrowitz et al. [14]:

$$BFR\;(\mathrm L/\min)\hspace{0.17em}=\hspace{0.17em}\lbrack Vmean\;(cm/min)\hspace{0.17em}\times\hspace{0.17em}\mathrm A\mathit\;{(cm)}^2\hspace{0.17em}\times\hspace{0.17em}60\rbrack/1000$$

where *Vmean*: the mean blood flow velocity of the jugular vein; *A*: the vein area.

To determine CI of the jugular vein, the following equation was used [38]:

$$\mathrm{CI}\;(cm.sec)\hspace{0.17em}=\hspace{0.17em}\mathrm A\;{(cm)}^2\;/\;\mathrm Vmean\;(cm/sec)$$

### Statistical analysis

Firstly, all obtained data were saved on an electronic spreadsheet (Excel® 2016, Microsoft® for Windows). Then, data were statistically analyzed using SPSS software (IBM SPSS® analytical program for Windows, 21), and presented as means ± SD. The normality of all obtained data was tested using the Kolmogorov–Smirnov test. The mean and SD of each vein measurement were calculated from three separate images, and these data were used for determination of the normal reference values of the jugular vein. Reference values were defined as mean ± 2 SD. Repeatability was calculated by repeated-measures analysis of variance using individual measurements obtained over 3 days and calculation of interclass correlation coefficients (ICC) [39]. Reproducibility of the daily mean measurements was tested using Student's t-test. To estimate the effects of sex and body condition score (BCS) on the ultrasonographic measurements, analysis of variance (ANOVA) was done. To assess the changes in different ultrasonographic variables between the left and right sides, a paired-sample t-test was carried out, as well as Bland–Altman analysis was conducted. For all statistical analyses, data were considered significant at *P* < 0.05.

## Data Availability

The datasets generated and/or analyzed during the current study are available from the corresponding author on reasonable request.

## Abbreviations

PT: Prothrombin time
APTT: Activated partial thromboplastin time
TD: Transverse diameter
D: Depth
SWT: Superficial wall thickness
DWT: Deep wall thickness
LD: Longitudinal diameter
PRF: Pulse repetition frequency
Vmean: Mean blood flow velocity
BFR: Blood flow rate
CI: Congestion index
ICC: Interclass correlation coefficients
BCS: Body condition score
